# Supplementary material for: The impact of pediatric early warning score and rapid response algorithm training and implementation on interprofessional collaboration in a resource-limited setting
Source: PLoS One. 2022 Jun 22;17(6):e0270253. doi: 10.1371/journal.pone.0270253 (PMC9216488; doi:10.1371/journal.pone.0270253)
Supplement: S1 Survey — (DOCX) [file pone.0270253.s001.docx]

**Collaboration and Communication in Patient Care**

**Pre-Implementation of PEWS-RL- Nurse Survey**

The statements below are related to the collaboration and communication between physicians and nurses. Please circle the number that best represents your opinion about the team process.*This questionnaire is not mandatory and by filling it out you give your consent for approval for analysis and publication of the data. All data is anonymous. This is not part of any evaluation.*

Nous aimerions connaitre votre opinion sur la collaboration et la communication dans notre équipe. Les déclarations ci-dessous sont en rapport avec la collaboration et la communication entre les médecins et les infirmièr(e)s de votre service. *Ce questionnaire est complètement anonyme n’est pas obligatoire. En le remplissant, vous donnez autorisez l’équipe de recherche d’analyser et de publier les données. Vos réponses ne font pas partie d’une évaluation.*

1. Physicians share all information with the nurses when making decisions on patient care Les médecins partagent toutes les informations avec les infirmières lors de la prise de décision concernant les soins aux patients

1 2 3 4 5

*Strongly Somewhat Neutral Somewhat Strongly*

*Disagree Disagree Agree Agree*

1. Decision-making responsibilities for patients are shared among nurses and physicians

Les responsabilités de prises de décisions sont partagées entre les infirmièr(e) et les médecins.

1 2 3 4 5

*Strongly Somewhat Neutral Somewhat Strongly*

*Disagree Disagree Agree Agree*

1. Nurses and physicians round together to share patient care information

Les médecins et les infirmièr(e) font le tour de salle ensemble pour partager les informations sur la prise en charge des patients.

1 2 3 4 5

*Strongly Somewhat Neutral Somewhat Strongly*

*Disagree Disagree Agree Agree*

1. My opinion is valued by my colleagues *(physicians, charge nurses, matron)* when communicating about my patient Mes collègues (médecins, infirmiers en chef, matrones) apprécient mon opinion lorsque je parle de mon patient

1 2 3 4 5

*Strongly Somewhat Neutral Somewhat Strongly*

*Disagree Disagree Agree Agree*

1. I feel the physicians listen and respond to me when I communicate my concerns regarding patient care Les medecins m’ecoutent quand je leur parle de mes preocupations concernant la prise en charge de mes paeints

1 2 3 4 5

*Strongly Somewhat Neutral Somewhat Strongly*

*Disagree Disagree Agree Agree*

1. When I feel there is a error made by the physician (verbal or written order) I feel comfortable notifying that physician when error is identified Je me sens a l’aise d’informer le medecin quand j’ai l’impression qu’il y a une erreur dans un ordre ou dans la prise en charge d’un patient

1 2 3 4 5

*Strongly Somewhat Neutral Somewhat Strongly*

*Disagree Disagree Agree Agree*

1. On my ward physicians and nurses work together as a team to monitor and assess patients Dans mon unité, les médecins et les infirmièr(e) travaillent ensemble en équipe pour prendre soin des patients.

1 2 3 4 5

*Strongly Somewhat Neutral Somewhat Strongly*

*Disagree Disagree Agree Agree*

1. How many times a day do nurses and physicians communicate with each other regarding a patient (Please check off one box)

Combien de fois par jour est-ce que les médecins et les infirmièr(e) de votre unité communiquent à propos d’un patient (Veuillez cocher une case)

0-1  2-3  4-5  6-7  7+

List 3 things that you think is the role of the nurse; Citez 3 choses que vous pensez sont le role de l’infimiere :

1.)_______________________________________________________________________________

2.)_______________________________________________________________________________

3.)_______________________________________________________________________________

List 3 things that you think is the role of the physician; Citez 3 choses que vous pensez sont le role du medecin:

1.)_______________________________________________________________________________

2.)_______________________________________________________________________________

3.)_______________________________________________________________________________

Any additional comments on how you feel nurses are respected by physicians when communicating about patient care; Avez-vous d’autres commentaires sur sur la façon dont les infirmières sont respectées par les médecins lorsque ces deux personnes parlent de la prise en charge des patients?

_______________________________________________________________________________________________________________________________________________________
