# Supplementary material for: The impact of pediatric early warning score and rapid response algorithm training and implementation on interprofessional collaboration in a resource-limited setting
Source: PLoS One. 2022 Jun 22;17(6):e0270253. doi: 10.1371/journal.pone.0270253 (PMC9216488; doi:10.1371/journal.pone.0270253)
Supplement: S4 Survey — (DOCX) [file pone.0270253.s004.docx]

The statements below are related to the collaboration and communication between physicians and nurses. Please circle the number that best represents your opinion about the team process. *This questionnaire is not mandatory and by filling it out you give your consent for approval for analysis and publication of the data. All data is anonymous. This is not part of any evaluation.*

Nous aimerions connaitre votre opinion sur la collaboration et la communication dans notre équipe. Les déclarations ci-dessous sont en rapport avec la collaboration et la communication entre les médecins et les infirmièr(e)s de votre service. *Ce questionnaire est complètement anonyme n’est pas obligatoire. En le remplissant, vous donnez autorisez l’équipe de recherche d’analyser et de publier les données. Vos réponses ne font pas partie d’une évaluation.*

1. What is your current job title or profession (Please check off one box) Quel est votre titre actuel? (Veuillez cocher une case)

Pediatrician  Resident

2. Physicians share all information with the nurses when making decisions on patient care Les médecins partagent toutes les informations avec les infirmières lors de la prise de décision concernant les soins aux patients

1 2 3 4 5 *Strongly Disagree Somewhat Disagree Neutral Somewhat Agree Strongly Agree*

*Fortement en désaccord Un peu en désaccord Neutre Plutôt d’acorrd Tout a fait d’accord*

3.Decision-making responsibilities for patients are shared among nurses and physicians. Les responsabilités de prises de décisions sont partagées entre les infirmièr(e) et les médecins.

1 2 3 4 5 *Strongly Disagree Somewhat Disagree Neutral Somewhat Agree Strongly Agree*

*Fortement en désaccord Un peu en désaccord Neutre Plutôt d’acorrd Tout a fait d’accord*

4. Nurses and physicians round together to share patient care information. Les médecins et les infirmièr(e) font le tour de salle ensemble pour partager les informations sur la prise en charge des patients.

1 2 3 4 5 *Strongly Disagree Somewhat Disagree Neutral Somewhat Agree Strongly Agree*

*Fortement en désaccord Un peu en désaccord Neutre Plutôt d’acorrd Tout a fait d’accord*

5. My opinion is valued by my colleagues *(physicians, charge nurses, matron)* when communicating about my patient. Mes collègues (médecins, infirmiers en chef, matrones) apprécient mon opinion lorsque je parle de mon patient

1 2 3 4 5 *Strongly Disagree Somewhat Disagree Neutral Somewhat Agree Strongly Agree*

*Fortement en désaccord Un peu en désaccord Neutre Plutôt d’acorrd Tout a fait d’accord*

6. On my ward physicians and nurses work together as a team to care for patients. Dans mon unité, les médecins et les infirmièr(e) travaillent ensemble en équipe pour prendre soin des patients.

1 2 3 4 5 *Strongly Disagree Somewhat Disagree Neutral Somewhat Agree Strongly Agree*

*Fortement en désaccord Un peu en désaccord Neutre Plutôt d’acorrd Tout a fait d’accord*

7. Nurses inform the physicians in a timely manner regarding patient deterioration. Les infirmièr(e) informent en général les médecins à temps quand l’état d’un patient se détériore.

1 2 3 4 5 *Strongly Disagree Somewhat Disagree Neutral Somewhat Agree Strongly Agree*

*Fortement en désaccord Un peu en désaccord Neutre Plutôt d’acorrd Tout a fait d’accord*

8. Nurses are accurate in their assessment of patient status. Les infirmièr(e) sont précises dans leur évaluation de l’état des patients.

1 2 3 4 5 *Strongly Disagree Somewhat Disagree Neutral Somewhat Agree Strongly Agree*

*Fortement en désaccord Un peu en désaccord Neutre Plutôt d’acorrd Tout a fait d’accord*

9. When a nurse calls me regarding a patient they are worried about I always go and assess that patient Quand une infirmièr(e) m’appelle à propos d’un patient dont ils/elles sont inquiets, je vais toujours évaluer ce patient

1 2 3 4 5 *Strongly Disagree Somewhat Disagree Neutral Somewhat Agree Strongly Agree*

*Fortement en désaccord Un peu en désaccord Neutre Plutôt d’acorrd Tout a fait d’accord*

10. How many times a day do nurses and physicians communicate with each other regarding a patient (Please check off one box). Combien de fois par jour est-ce que les médecins et les infirmièr(e) de votre unité communiquent à propos d’un patient (Veuillez cocher une case)

0-1  2-3  4-5  6-7  7+

11. Do you feel decision making for patients is more collaborative since the implementation of the PEWS and Rapid Response Team program? (Please check off one box) Estimez-vous que la prise décision pour les patients est plus collaborative depuis la mise en œuvre du programme PEWS et de l’équipe d’intervention rapide? (Veuillez cocher une case)

1 2 3 4 5 *Strongly Disagree Somewhat Disagree Neutral Somewhat Agree Strongly Agree*

*Fortement en désaccord Un peu en désaccord Neutre Plutôt d’acorrd Tout a fait d’accord*

12. What is the nurses role in monitoring and assessing patients? Quel est le rôle des infirmièr(e) quant à la surveillance et à l’évaluation des patient?

_________________________________________________________________________

_________________________________________________________________________

13. Any additional comments on how you feel the implementation of PEWS has changed the way nurses and physicians communicate regarding patient care? Tout commentair supplémentaire sur la maniére dont vous pensez que la mise en œuvre de PEWS a changé la facon dont les infirmiéres et les médecins communiquent en ce qui concerne les soins aux patients ?

____________________________________________________________________________________________________________________________________________________________________________________________________________________________________
